# Supplementary material for: Curated and harmonised transcriptomics datasets of interstitial lung diseases
Source: Data Brief. 2025 Oct 14;63:112139. doi: 10.1016/j.dib.2025.112139 (PMC12581653; doi:10.1016/j.dib.2025.112139)
Supplement: Supplementary file 1 [file mmc1.zip › Supplementary_material/RNA-seq/fastQC reports/GSE184316/SRR15926757_fastqc.html]

SRR15926757.fastq.gz FastQC Report 

FastQC Report

Mon 5 Feb 2024  
SRR15926757.fastq.gz

## Summary

- Basic Statistics
- Per base sequence quality
- Per sequence quality scores
- Per base sequence content
- Per sequence GC content
- Per base N content
- Sequence Length Distribution
- Sequence Duplication Levels
- Overrepresented sequences
- Adapter Content

## Basic Statistics

| Measure | Value |
| --- | --- |
| Filename | SRR15926757.fastq.gz |
| File type | Conventional base calls |
| Encoding | Sanger / Illumina 1.9 |
| Total Sequences | 6261712 |
| Sequences flagged as poor quality | 0 |
| Sequence length | 25-386 |
| %GC | 49 |

## Per base sequence quality

## Per sequence quality scores

## Per base sequence content

## Per sequence GC content

## Per base N content

## Sequence Length Distribution

## Sequence Duplication Levels

## Overrepresented sequences

| Sequence | Count | Percentage | Possible Source |
| --- | --- | --- | --- |
| GGAAGACAGTGTGGCGATTCCTCAGGGATCTAGAACTAGAAATACCATTG | 55462 | 0.8857322086994739 | No Hit |
| GAAGGACCTCTTCAAGGAGAACTACAAACCACTGCTCAAGGAAATAAAAG | 46812 | 0.7475910741343582 | No Hit |
| ATTGATTCTTCCTACCCATGAGCATGGAATGTTCTTCCATTTGTTTGTAT | 43209 | 0.6900508998178134 | No Hit |
| ATAGCAGCATGATTTATAGTCCTTTGGGTATATACCCAGTAATGGGATGG | 34562 | 0.5519576754727781 | No Hit |
| GAAGGACCTCTTCAAGGAGAACTACAAACCACTGCTCAATGAAATAAAAG | 29239 | 0.4669489749768114 | No Hit |
| AGGCTACACACTGAGGACTCTGTTCCTCCCCTTTCCGCCTAGGGGAAAGT | 21176 | 0.33818227347409147 | No Hit |
| TTACAAAAGCAGAATAAAAGCGAAAAGAAATGAAAATGTTACACTACATT | 19630 | 0.313492540059332 | No Hit |
| CAATCCACTTGATCCCAACTCATCTCTCATTTATTTCGGCTTCTTTTATT | 17454 | 0.27874166042769133 | No Hit |
| ATAGCAGCATGATTTATAATCCTTTGGGTATATACCCAGTAATGGGATGG | 16838 | 0.26890409523785186 | No Hit |
| TGATATGGTTCCTGGCAAGCCCATGTGTGTTGAGAGCTTCTCAGACTATC | 16732 | 0.2672112674616782 | No Hit |
| CAGTGGGGCCACGAGCTGAGTGCGTCCTGTCACTCCACTCCCATGTCCCT | 16416 | 0.26216472427987747 | No Hit |
| TGATGACACCCACCGCAACTGTCTGTCTCATATCACGAACAGCAAAGCGA | 15815 | 0.25256671019043997 | No Hit |
| GGGAAGCGGGGAATGTCTACGTGCGTATGCACGTGGCACTCTCTGCCCGA | 15705 | 0.25081000212082577 | No Hit |
| TTCAGCCCTGATCAAGACATGAGGGAGGCAGGGGCTCAGCTGAAGAAGCT | 14000 | 0.22358102704180582 | No Hit |
| CACCTCCCCGAAGCTCAGGGAGAGCCCTGTTAGGGCCGCCTCTGGCCCTA | 13652 | 0.21802344151248093 | No Hit |
| GGCAATGAGCGGTTCCGCTGCCCTGAGGCACTCTTCCAGCCTTCCTTCCT | 11450 | 0.1828573399734769 | No Hit |
| GTCCACGTCACACTTCATGATGGAGTTGAAGGTAGTTTCGTGGATGCCAC | 10705 | 0.17095963532018082 | No Hit |
| ATTGATTCTTCCTACCCATGAGCATGGAATGTTCTTCCATTTGTTTGTGT | 10660 | 0.170240982018975 | No Hit |
| TACCACTGCCACCTTCTCCATCGGCTCCACTGGCCTCGTGGTGTATGACT | 10300 | 0.16449175560932858 | No Hit |
| TTTTCCATGAGCTTAATGATGCTTTCTCTGGGCTTTTGGGGGAGGGTGTC | 9752 | 0.15574015540797786 | No Hit |
| GGCATCAAAAGTGATGGACTGCCCATTGCTGGAGAAGACCTTCTCTCCTA | 9612 | 0.1535043451375598 | No Hit |
| CTTCATGATGTAGCAGCAGGTGCCAGGGGCTGGCTTGTAGGCGATCAGCA | 9485 | 0.15147614582082344 | No Hit |
| GGAAGACAGTGTGGCGATTCCTCAAGGATCTAGAACTAGAAATACCATTG | 9068 | 0.14481662522964964 | No Hit |
| GGCCATTTTCATGATATTGATTCTTCCTACCCATGAGCATGGAATGTTCT | 8367 | 0.13362160380419924 | No Hit |
| AGACATCAAATCCTGCAGACAAGGGGAGCCCTCAGTCTGCAGGGCTCCAT | 7983 | 0.1274890956339097 | No Hit |
| GCATGTGTCTTTATAGCAGCATGATTTATAGTCCTTTGGGTATATACCCA | 7785 | 0.12432702110860415 | No Hit |
| GGAAGACAGTGTGGTGATTCCTCAGGGATCTAGAACTAGAAATACCATTG | 7196 | 0.1149206478994882 | No Hit |
| CATATGGCTCTCCCAGAGGCAGAAGGACAATGGCTGTTTCAGGAGCTCTG | 6957 | 0.11110380036641737 | No Hit |
| ACAAGAAGTACATCAAAGATTACATGAAATCAATCAAAGGGAAACTTGAA | 6926 | 0.11060872809225336 | No Hit |
| AGAAGTCACTGATCAGACACACTAGTGTGGCCTTGTTGGCTTGGAGCTCC | 6824 | 0.10897978060952021 | No Hit |
| ATAGCAGCATGATTTATAGTCATTTGGGTATATACCCAGTAATGGGATGG | 6486 | 0.1035818958137966 | No Hit |

## Adapter Content

Produced by FastQC (version 0.11.7)
